# Review of CAR T-Cell Therapy in Multiple Myeloma: A Canadian Perspective

**DOI:** 10.3390/curroncol31070292

**Published:** 2024-07-06

**Authors:** Steven Chun-Min Shih, Sita Bhella

**Affiliations:** Department of Medical Oncology and Haematology, Princess Margaret Cancer Centre, Toronto, ON M5G 2M9, Canada; steven.shih@uhn.ca

**Keywords:** myeloma, immunotherapy, CAR T

## Abstract

Multiple myeloma (MM) is an incurable plasma cell malignancy. In the context of the current standard of care therapies in Canada, outcomes among patients with relapsed/refractory multiple myeloma (RRMM), particularly those with triple-class (or more) refractory disease remain poor. Immunotherapies have significantly changed the treatment landscape of MM. Since 2021, two BCMA-targeting CAR T-cell therapy products have been approved for RRMM—namely Idecabtagene vicleucel (Ide-cel) (ABECMA^®^) and Ciltacabtagene autoleucel (Cilta-cel) (CARVYKTI^®^), both of which are available in the US and Europe. Although they have shown unprecedented efficacy in RRMM, their clinical and logistical limitations must be acknowledged. MM CAR T-cell therapy is likely to be approved in Canada soon. Therefore, it is timely that we review the latest evidence for commercially available CAR T-cell therapy in multiple myeloma, with a focus on its relevance and impact in the Canadian setting. There will be challenges to access and strategies must be in place to ensure equitable care for all Canadians with MM. Alongside haematologists working in the immune effector cell therapy programs, providers in the community will also play a role in the ongoing monitoring and management of long-term side effects including opportunistic infections and late neurotoxicity.

## 1. Introduction

Multiple myeloma (MM) is a plasma cell malignancy characterized by an abnormal increase in monoclonal paraproteins and can result in anaemia, hypercalcaemia, renal dysfunction, and bone lytic lesions [1]. It is the second most common haematologic malignancy, with the highest incidence rates in North America, Europe, and Australasia [2].

Although considerable progress in therapies has been made over the last two decades, leading to improved outcomes with deeper responses and longer survival, multiple myeloma remains incurable, and most patients will eventually relapse [3]. Furthermore, outcomes among patients with relapsed/refractory multiple myeloma (RRMM), particularly those with triple-class (or more) refractory disease, remain poor [4,5,6].

Chimeric antigen receptor (CAR) T-cell therapy is a novel approach in which the patient’s own T cells are harvested and genetically modified to redirect against specific antigens on the surface of cancer cells [7]. Currently, CAR T-cell therapy is indicated and funded in Canada for third-line treatment for B-acute lymphoblastic leukaemia, large B-cell lymphoma, and mantle cell lymphoma. Since 2021, the US Food and Drug Administration (FDA) has approved two CAR T-cell therapy products for RRMM, namely Idecabtagene vicleucel (Ide-cel) (ABECAMA^®^) and Ciltacabtagene autoleucel (Cilta-cel) (Carvykti^®^), both of which are available in the US and Europe. Although they have shown unprecedented efficacy in RRMM, their clinical and logistical limitations must be acknowledged [8,9].

CAR-T cell therapy for RRMM is likely to be approved in Canada soon. Therefore, it is timely that we review the latest evidence for CAR T-cell therapy in multiple myeloma, with a focus on its relevance, challenges, and impact in the Canadian setting.

## 2. A Population in Need

There have been key studies which consistently demonstrate a poor prognosis in patients with triple-refractory disease, and they are summarized here (see Table 1).

The MAMMOTH study was a multicentre, retrospective study evaluating the natural history and outcomes of 275 US patients from 14 academic centres, with active MM and refractory to anti-CD38 monoclonal antibody. Most of these patients were also refractory to lenalidomide, pomalidomide, and bortezomib. At least half of the patients were considered triple-refractory or quad-refractory, whilst a quarter of patients were penta-refractory. Overall, the median overall survival (mOS) of patients refractory to anti-CD38 monoclonal antibody was 8.6 months, whilst the mOS for the cohort of penta-refractory patients was 5.6 months. The median progression free survival (mPFS) of those who received at least one subsequent line of therapy was a dismal 3.4 months, with objective responses seen in less than half of those patients [6].

The LocoMMotion study was a multinational, prospective observational study evaluating the use of real-life current standard of care therapies in US and European patients with RRMM who have received at least three prior lines of therapy or were double-refractory to a proteasome inhibitor (PI) and an immunomodulatory drug (IMiD), or triple-refractory to PI, IMiD, and anti-CD38 monoclonal antibody, and have documented progression during or after their last line of therapy. The overall response rate was 29.8% with median duration of response at 7.4 months. The mPFS was 4.6 months and the mOS was 12.4 months [4].

Visram et al. from the Canadian Myeloma Research Group (CMRG) conducted a large multicentre retrospective cohort study analysing the real-world outcomes of Canadian patients with multiple myeloma relapsing on anti-CD38 monoclonal antibody regimens [5]. The most common standard of care regimens used after relapse on index anti-CD38 monoclonal antibody were PI/steroid doublet, followed by either a combination of PI or IMiDs with an alkylator (most commonly cyclophosphamide). Overall, the mPFS from the start of subsequent therapy was 4.6 months, and the mOS was 13.3 months [5]. Patients with triple-class refractory disease constituted 58% (199/346) of this study cohort; their mPFS and mOS were 4.4 months and 10.5 months, respectively. These are worse outcomes compared to those who did not have triple-class refractory disease, with mPFS and mOS at 6.0 and 17.5 months, respectively [5].

It should be noted that none of the patients in the aforementioned studies had received CAR T-cell therapy or a bispecific antibody by the study analysis cutoff date [4,5,6]. This highlights the clear unmet needs of patients with triple-class refractory MM in the context of the currently available “standard of care” regimens available in Canada. There is compelling evidence that the newer immunotherapeutic approaches including CAR T-cell therapy will improve on the current Canadian real-world benchmarks.

## 3. CAR T-Cell Therapy: Practical Basics

Both Ide-cel and Cilta-cel are autologous, second-generation CAR T-cell therapies that target the B-cell maturation antigen (BCMA). BCMA is an excellent target as it is ubiquitously present on the surface of plasma cells including MM cells. It is expressed at much lower levels in other haematologic cells and absent in non-haematologic tissues [10]. There are, however, structural differences between the two CAR T-cell products. Ide-cel has a single murine scFv binding domain for the BCMA antigen, whilst Cilta-cel has two BCMA-binding domains [7].

Preparation involves leukapheresis of a patient’s T cells, followed by CAR T-cell manufacturing in a good manufacturing processes facility which takes approximately 4–6 weeks. During this period, patients may require bridging therapy for disease control. Once the manufacturing stage has been completed, patients will undergo lymphodepletion (typically with fludarabine and cyclophosphamide), followed by CAR T-cell infusion [3,7]. To manage the adverse events and toxicities of CAR T-cell therapy, including cytokine release syndrome (CRS), immune effector cell-associated neurotoxicity syndrome (ICANS), severe cytopenia, and infections, most centres require inpatient hospitalization after infusion, though outpatient CAR T-cell service can be delivered in highly selected patients in select centres [7].

CRS refers to a systemic inflammatory response due to the release of cytokines from cells and is characterized by fever, tachypnoea, headache, tachycardia, hypotension, rash, or hypoxia. ICANS refers to a disorder of the central nervous system following any immunotherapy that can result in the activation or engagement of endogenous or infused T cells or other immune effector cells. Symptoms or signs can be progressive and include altered handwriting, aphasia, altered level of consciousness, impairment of cognitive skills, motor weakness, seizures, and cerebral oedema. There are consensus grading systems for both syndromes which management protocols are based upon [11]. Treatment includes supportive care, steroids, and anti-IL6 and/or anti-IL1 agents [3]. High-grade cytopenias are also common for which transfusion support and growth factors may be required [3,7]. Both the lymphodepleting conditioning regimens and BCMA CAR T-cell therapy leads to significant immune suppression and elevated risks of acquiring serious infections such as pneumonias and CMV reactivation. BCMA-directed CAR-T therapy depletes both malignant and normal plasma cells, leading to severe hypogammaglobulinaemia. Overall, the infection risk remains the greatest during the first few months post CAR T-cell infusion, though hypogammaglobulinaemia may persist for longer. Many experts recommend starting IVIG (0.4 g/kg/month) when IgG levels drop below 400 mg/dL for infection prophylaxis [8,9], though there are no prospective data to guide such practice.

For at least the first 30 days, there is a need to reside within a specified travel distance of the treatment centre to ensure safety and timely access to specialized healthcare post CAR T-cell infusion. Furthermore, patients are not allowed to drive for the first eight weeks post-infusion due to the risk of ICANS. Therefore, all patients will require at least a reliable caregiver who plays a key role in support, day-to-day care, the monitoring of adverse events, and providing timely transport back to the hospital for urgent treatment [12].

Apart from BCMA-specific CAR T-cell therapy, other BCMA-targeted therapy (BCMA-TT) includes antibody–drug conjugates (ADCs), belantamab mafodotin, and bispecific T-cell engagers (BiTEs)—teclistamab and elranatamab [13]. It should be noted that discussions on ADC and BiTE therapies are beyond the scope of this review.

## 4. Idecabtagene Vicleucel (Ide-cel): In Trial and Real World

On 26 March 2021, Ide-cel was the first myeloma CAR T-cell therapy to be approved by the FDA [8], and this was based on the pivotal KarMMA trial [14] (see Table 2). This was a single-group, phase II study evaluating the efficacy and safety of Ide-cel in patients with RRMM who received at least three prior regimens including an IMiD, PI, and anti-CD38 monoclonal antibody. Of the 128 patients who received Ide-cel, their median number of prior lines of therapy was six, 84% (108/128) had triple-class refractory disease, whilst 26% (33/128) were penta-refractory. The overall response rate (ORR) was 73% (94/128), and 33% (42/128) achieved a complete response (CR) or better at a median follow-up of 13.3 months [14]. Furthermore, of the participants who achieved a CR or better, 79% (33/42) of patients achieved an MRD negative status at a sensitivity level of 10-5. The median time to first response was 1.0 months, whilst the median time to a CR or better was 2.8 months. Overall, the mPFS was 8.8 months, and those who achieved a CR or better had an mPFS of 20.2 months. The estimated mOS was 19.4 months, with an overall survival of 78% at 12 months [14]. Of note, a high incidence of response was consistently observed in most subgroups including older patients, those who had bridging therapy, more aggressive disease features, and high-risk cytogenetic abnormalities, triple or penta-refractory disease, high tumour burden, and extramedullary disease [14].

In regard to adverse events, almost all patients had a Grade 3 or 4 event. Haematologic toxicity was the most common adverse event with 91% neutropenia, 70% anemia, and 63% thrombocytopenia. Grade 3 or 4 haematologic toxicity was also common—89% neutropenia, 60% anemia, and 52% thrombocytopenia. Infections occurred in 69% of patients and were Grade 3 or higher in 22% of patients. The use of antimicrobial agents, growth factors, and immunoglobulins was common. Cytokine release syndrome occurred in 84% and was mostly Grades 1 or 2. Only 5% had Grade 3 or 4 CRS. The median time to CRS onset was 1 day. Neurotoxicity occurred in 18% of patients and only 3% were Grade 3 or higher [14]. The median onset time for neurotoxicity was 2 days. It should be noted that the report does not differentiate between ICANS and non-ICANS. Of note, a total of 44 patients (34%) died during the study, with most deaths [21] relating to disease progression. In total, three patients (2%) died within 8 weeks of Ide-cel infusion due to Ide-cel related adverse events (bronchopulmonary aspergillosis, gastrointestinal bleed and cytokine release syndrome) and one patient (1%) died between 8 weeks and 6 months from cytomegaloviral pneumonia [14].

KarMMa-RW was a global, noninterventional retrospective study which compared the outcomes of the KarMMa study cohort against real-world outcomes from patients (derived from large registries) with RRMM who had received at least three prior lines of therapy and would have met the inclusion criteria of the KarMMa study but only received the standard of care treatment. Jagannath et al. [22] reported a significantly improved ORR: 76.44% in the KarMMa cohort vs. 32.0% in the eligible RRMM cohort. The VGPR or better rate was 58.7% in the KarMMa cohort compared with 13.7% in the eligible RRMM patients. The mPFS was significantly prolonged in KarMMa participants when compared with eligible RRMM patients (11.6 months vs. 3.5 months) at a median follow-up of 12.9 months and 11.1 months, respectively. The median OS was significantly improved with Ide-cel in KarMMa participants versus the eligible RRMM cohort—20.2 months vs. 14.7 months, respectively (hazard ratio 0.45), at a median follow-up of 14.4 months and 15 months, respectively. Overall, this study demonstrated a clear benefit with Ide-cel treatment over the available therapies at the time. Of note, of the real-world patients in this study, 94 different treatment regimens were used, which highlights the lack of standard of care therapy for triple-class exposed RRMM [22]. It should be noted, however, that there are limitations with the methodology of this study including unmeasured confounders that could not be controlled. Furthermore, some baseline prognostic characteristics were not balanced between the two groups, and there were significant missing data in the eligible RRMM cohort.

Delforge et al. [23] reported on health-related quality of life (HRQoL) outcomes in the KarMMa trial. They noted that the baseline burden of RRMM was high among these patients at enrolment. The mean baseline QLQ-C30 scores were meaningfully worse alongside those of the re-weighted general population. Patients receiving Ide-cel treatment reported a meaningful improvement in all primary HRQoL analyses, as early as 1 or 2 months. Improvements were generally sustained over time, however, there were decreasing sample sizes by 12 months through to 18 months, and this introduces selection bias, favouring those who have had a treatment response and are still alive for follow-up. There were statistically and clinically meaningful improvements in QLQ-C30 measures of pain, physical functioning by 1 month, fatigue, cognitive functioning, and global health status by 2 months. Fatigue, pain, and physical functioning improvement were sustained through 18 months after Ide-cel. Cognitive functioning remained stable, with statistically significant and clinical improvement from 2 months to 9 months [23].

Delforge et al. [24] subsequently reported on the longitudinal patient experience outcome on 45 (35%) of the 128 KarMMA participants who received an Ide-cel infusion. As part of the KarMMA trial, post-infusion interviews were conducted at regular intervals from 6 to 24 months post Ide-cel infusion. The main advantages perceived by patients were related to efficacy and the avoidance of other treatments such as chemotherapy. Most patients (42/45, 93%) reported that the initial efficacy benefit was an advantage. In total, 34/45 (76%) were generally satisfied with their treatment response. Half of the patients (23/45, 51%) reported improved well-being, particularly physical benefits including energy levels. Most patients expressed that the advantage was avoiding other treatment or maintenance therapies (34/45, 76%) or other treatments (21/45, 47%) [24]. Overall, these findings should be interpreted with caution given the high attrition rate through the 24-month follow-up period and that the participants agreed to the interview in this sample. This again results in selection bias, favouring those who have had a treatment response, are still alive for follow-up, and well enough for the interview. On the other hand, the perceived disadvantages include side effects post-infusion (27/45, 60%), the eventual lack of efficacy (23/45, 51%) which was primarily due to the lack of a durable response, and lingering or persistent side effects still present at 6 months onwards (18/45, 40%). Many patients identified the post-infusion monitoring process as a disadvantage (24/45, 33%) [24].

Hansen et al. [15], from the Myeloma CAR-T Consortium (see Table 2), conducted a retrospective multicentre observational study of patients planned for standard of care Ide-cel for US patients with RRMM who had received at least four prior lines of therapy. In total, 196 patients completed leukapheresis with intent to manufacture. A total of 12 patients had a manufacturing failure (6%), but 7 of these were manufactured successfully with repeat apheresis. In total, 20 patients were pending infusion at data cutoff; hence, 159 patients received Ide-cel. Of note, 129 (75%) patients in this study cohort would have been ineligible for the stringent inclusion/exclusion criteria of the KarMMa trial, with the most common reasons being inadequate organ function, prior use of BCMA-targeting therapy, cytopenias, and an ECOG score of at least two or more. Furthermore, this real-world cohort (cf. to the KarMMa trial) had more patients with extramedullary and penta-refractory disease at 48% vs. 39% and 44% vs. 26%, respectively [15]. The best ORR and CR (or better) rates were 84% and 42%, respectively. Of the patients who achieved at least a CR, 72% achieved an MRD negative status at a sensitivity of 10-5 [15]. At a median follow-up of 6.1 months, the mPFS and mOS were 8.5 months and 12.5 months, respectively. Overall, the efficacy of Ide-cel in this real-world report was comparable to those reported in the KarMMA study.

In the Hansen et al. cohort, CRS of any grade occurred in 82% of patients whilst Grade 3 or higher occurred in 3% of patients. Any grade of neurotoxicity was seen in 18%, whilst Grade 3 or higher occurred in 6% of patients. A total of 8% patients required transfer to an intensive care unit. Any grade of neutropenia, anaemia, and thrombocytopenia occurred in 97%, 95%, and 95%, respectively, whilst Grade 3 or higher neutropenia, anaemia, and thrombocytopenia occurred in 88%, 51%, and 95%, respectively. Grade 3 or higher haematologic toxicity lasting 30 days or more after infusion included neutropenia in 60%, anemia in 38%, and thrombocytopenia in 59% of patients [15]. In total, 74% of patients required granulocyte colony-stimulating factor (GCSF), 15% received a thrombopoietin agonist, and 5% received an autologous stem cell boost. Infections occurred in 34% of patients, primarily bacterial and viral, though two patients had a fungal infection. A total of 30 (19%) patients died by the last follow-up and 20 patients were attributed to disease progression. Of these, two had an unknown cause of death. Of the remaining eight, three had Ide-cel-related toxicity of which two had Grade 5 CRS and one had hemophagocytic lymphohistiocytosis. Three had COVID-19 disease related mortality and two had cardiomyopathy [15]. Overall, the safety profile and rates of CRS, neurotoxicity, infection, and cytopenias in this real-world cohort were comparable to those in the KarMMa trial.

More recently, Sidana et al. [16] reported on the safety and efficacy of Ide-cel from the CIBMTR registry (see Table 2). Of the 603 patients infused with Ide-cel, the median PFS at 6 months was 62% and the median OS at 6 months was 82%. These are favorable despite a very heavily pretreated population, and many would have also been ineligible for the KarMMa clinical trial [16].

Overall, these real-world studies alongside the original KarMMa trial support the efficacy of Ide-cel for patients with RRMM with a manageable safety profile.

## 5. Ciltacabtagene Autoleucel (Cilta-cel): In Trial and Real World

On 28 February 2022, Cilta-cel was the second CAR-T therapy to be approved for RRMM and this was based on the pivotal CARTITUDE-1 trial in the United States (see Table 2).

CARTITUDE-1 was a single-arm open-label phase Ib/II study which investigated the use of Cilta-cel for RRMM at 16 US centres. Eligible patients were those with RRMM as per IMWG criteria who had received at least three prior regimens or were double-refractory to IMiD and PI, and had received an IMiD, PI, and an anti-CD38 monoclonal antibody [17]. In total, 113 patients were enrolled in this study and all underwent apheresis, but 16 patients did not receive Cilta-cel infusion and the reasons included disease progression, death, or study withdrawal. There was no patient discontinuation due to manufacturing failure [17]. Of the 97 remaining patients who received Cilta-cel infusion, 24% had high-risk cytogenetics, 13% had extramedullary disease, 88% had triple-class refractory disease, and the median number of prior therapies was six. At a median follow-up of 12.4 months, the ORR was 97% and 67% achieved a stringent CR. The median time to first response was 1 month. The median duration of response was not reached. The mPFS was not reached. The overall PFS and OS at 12 months were 77% and 89%, respectively [17].

In the CARTITUDE-1 trial, haematologic adverse events were the most common. Grade 3–4 neutropenia was observed in 95%, anemia in 68%, leukopenia in 61%, thrombocytopenia in 60%, and lymphopenia in 50% of participants. Infections occurred in 58% of patients, of which 20% were Grade 3–4. Upper respiratory tract infection was the most common infection overall, whilst pneumonia and sepsis were the most common Grade 3–4 infections [17].

Furthermore, in the CARTITUDE-1 trial, a CRS was common, occurring in 95% of patients; however, only 4% had Grade 3–4 CRS. The median time to onset of CRS was 7 days, with a median duration of 4 days. There was one case of Grade 5 CRS and HLH. Neurotoxicity occurred in 20 (21%) of participants, with 9% experiencing Grade 3 or higher neurotoxicity [17]. In total, 16 (17%) patients had ICANS, of which only 2 patients were Grade 3–4. The median time to ICANS was 8 days and the median duration was 4 days. Other neurotoxicities (such as cranial nerve palsies and parkinsonism) occurred in 12 (12%) of patients and the median time to onset was 27 days. Neurotoxicity symptoms were variable, though five patients had a cluster of movements and neurocognitive treatment-emergent adverse events. Six patients recovered from neurotoxicity event with a median recovery time of 74.5 days. One participant died from Grade 5 neurotoxicity [17].

Martin et al. [18] reported the 2-year follow-up of the CARTITUDE-1 study. In total, 66 of the 97 patients remained on the study. At a median follow-up of 27.7 months, the ORR was 97.9% and the sCR rate was 82.5%. At 27 months, the PFS and OS rates were 54.9% and 70.0% respectively. Since the initial report, there was one new case of parkinsonism, leading to total of six on the CARTITUDE-1 study, but no new cases of CRS. By this 2-year follow-up, 30 deaths occurred during the study, 14 of which were due to disease progression and 6 were treatment-related deaths. The remaining deaths were not related to study treatment. In total, 20 secondary primary malignancies (SPM) were reported in 16 patients, but none were deemed related to Cilta-cel [18].

A subsequent follow-up analysis of the CARTITUDE-1 trial reported at a median follow-up of 33.4 months that the median duration of response was 33.9 months, the median PFS was 34.9 months, and the mOS was not reached. It was estimated that there was 62.9% survival at 36 months [19]. Of the 49 MRD evaluable patients, 26 had MRD negativity (at sensitivity of 10^−5^) sustained for ≥12 months, of which 20 sustained MRD negative ≥ CR. The mPFS was not reached in these patients. There were no new safety signals or neurotoxicity events [19].

Martin et al. also reported on patients’ HRQoL for the CARTITUDE-1 trial [25]. Of note, there was an initial decrease at Day 7 for global health status (GHS), physical functioning, role functioning, social functioning, fatigue, and nausea or vomiting. This was consistent with the potential onset of adverse events associated with CRS. However, after this, there were improvements in GHS, physical functioning, and emotional functioning scales and decreases in symptom-based scores. These improvements were maintained over time with a similar trajectory to Ide-cel. This supports the general tolerability of Cilta-cel.

Hansen et al. [20] conducted a retrospective study evaluating the efficacy and safety of Cilta-cel in standard of care settings (see Table 2). By the study cutoff date (31 December 2022), 177 patients underwent leukapheresis from 1 of the 12 US academic centres, of which 139 received Cilta-cel infusion. Of note, this cohort had more high-risk disease with 35% of patients having extramedullary disease (cf. to 13% in CARTITUDE-1) and 41% with high-risk cytogenetics (cf. to 24% in CARTITUDE-1). Of note, 55% of patients in this cohort would not have been eligible for CARTITUDE-1. CRS was seen in 81% (≥Grade 3: 7%) and ICANS in 22% (≥Grade 3: 8%). Delayed neurotoxicity was seen in 9% (cranial nerve palsy in eight, parkinsonism in two, others in three patients). Grade ≥ 3 cytopenias at Day ≥ 30 were seen in 75% of patients. Infection occurred in 32% of patients. By Day 30, the best response rates were as follows: 80% achieved at least a PR, 62% achieved at least a VGPR, and 40% achieved a CR. In total, 17 patients had died: 4 were due to disease progression and 13 (9%) due to treatment-related adverse events (Grade 5 CRS in three, infection in six, CRS/infection in one, ICANS in one, and delayed neurotoxicity in two). Overall, patients still had a favourable overall response despite more patients having high-risk features, though the ORR and CR rates were somewhat lower than reported in the CARTITUDE-1 trial. Due to the short-term follow-up, PFS/OS data are not available. The results from longer-term follow-ups are awaited [20].

Similarly, real-world reports of Cilta-cel alongside reports of the CARTITUDE-1 trial support the efficacy and safety of Cilta-cel for patients with RRMM.

## 6. Comparison of Cilta-cel and Ide-cel

Comparison of Cilta-cel and Ide-cel in terms of efficacy and toxicity is challenging, owing to the lack of head-to head prospective trials. Attempts have been made to compare the two.

Martin et al. [26] conducted a matching-adjusted indirect comparison with individual patient data from the CARTITUDE-1 and KarMMa trials. The CARTITUDE-1 population was adjusted to match the eligibility criteria and distribution of prognostic factors in KarMMa. Infused patients from CARTITUDE-1 who satisfied the eligibility criteria of KarMMa were reweighted to adjust for imbalances in baseline characteristics of prognostic significance. In total, 15 potentially important prognostic factors, which were identified based on literature review and clinical expertise, were ranked in order of importance prior to conducting the analysis. Here, the effect of Cilta-cel for PFS was statistically significant and superior to Ide-cel after base case adjustment (HR 0.37). For OS, the estimated treatment effect was in favour of Cilta-cel (HR 0.55). Sensitivity analysis also favoured Cilta-cel over Ide-cel. In terms of base case adjustments, patients treated with Cilta-cel were 1.3-fold more likely to respond and 2.2-fold more likely to achieve at least a CR compared with Ide-cel. The duration of response suggested Cilta-cel was statistically significantly superior to Ide-cel [26]. However, these data need to be interpreted with caution as there may be unknown residual confounding variables and they are unable to be adjusted for when making a comparative analysis of non-randomised data.

Gill et al. [27] reported on the outcomes of 56 patients consecutively treated at a single US centre between 28 June 2021 to 3 July 2023. In total, 53 patients had evaluable responses and were included in the analysis. A total of 35 (66%) patients received Ide-cel, whereas 18 (34%) patients received Cilta-cel. Overall, treatment was well tolerated, with only one therapy-related mortality. The overall response rate for the entire cohort was 75.4% with a median PFS of 11.9. The mOS was not reached. The median ORR for Cilta-cel vs. Ide-cel was 94.% and 65.7%, respectively. The median PFS for Cilta-cel vs. Ide-cel was NR vs. 10.9 but not statistically significant. It should be noted the follow-up was very short for Cilta-cel. The authors noted a longer manufacturing time for Cilta-cel which introduced bias to favour using Ide-cel for patients with more aggressive/rapidly progressive disease, and thus would have negatively impacted the outcome for Ide-cel [27].

With respect to toxicity between the two agents, a later onset of CRS was seen with Cilta-cel at a median of 7 days [17] as opposed to 1–2 days with Ide-cel [14,16]. This may be due to a lower median CAR T-cell dose in the CARTITUDE-1 trial. The late onset of CRS may make Cilta-cel more amenable to outpatient administration.

Similarly, a later onset of neurotoxicity was seen in Cilta-cel as opposed to Ide-cel at a median of 8 days and 2 days, respectively [14,19,20]. In the KarMMa study, 18% of participants who received Ide-cel had any grade of neurotoxicity with only 3% developing Grade 3 neurotoxicity [14]. On the other hand, in the CARTITUDE-1 trial, 21% of participants who received Cilta-cel had any grade of neurotoxicity and 9% developed Grade 3 neurotoxicity [17].

Late onset neurotoxicity (including cranial nerve palsy, parkinsonism, peripheral neuropathies, and neurocognitive disorder) was seen in 12% of patients receiving Cilta-cel in the CARTITUDE-1 trial at a median onset of 27 days [17]. In total, six (6%) participants developed parkinsonism. Hansen et al. described a real-world cohort of patients treated with standard of care Cilta-cel and also reported delayed neurotoxicity at 9%, in which 1.4% of patients developed parkinsonism [20]. Although there were no reports of parkinsonism in the original KarMMa trial, parkinsonism has been described following Ide-cel infusion [21]. Overall, Ide-cel may be preferred in patients with underlying neurologic disease.

The choice between the products is difficult in the absence of a randomized prospective clinical trial, as both are effective with tolerable safety profiles [28]. It should be noted that often patients require timely access to CAR-T therapy, and hence access and product availability may become more important as a deciding factor.

## 7. Special Populations

### 7.1. Renal Impairment

Sidana et al. [29] conducted a retrospective multicentre observational study of patients with and without renal impairment, treated with standard of care Ide-cel from the 11 medical centres in the US multiple myeloma immunotherapy consortium. Renal impairment (RI) was defined as a creatinine clearance (CrCl) <50 mL/min at the time of CAR T-cell therapy and a CrCl of <30 mL/min was defined as severe RI. Of the 214 patients who received Ide-cel, 28 (13%) had renal impairment; and of these, 11 (39%) had severe renal impairment. One patient was on dialysis. Of note, the patients with renal impairment tended to be older, more likely to be female, and had a higher likelihood of having R-ISS Stage 3 disease. Patients with RI were more heavily pretreated with a median of eight lines of therapy compared to six prior lines of therapy in patients without RI. Dose reduction for fludarabine was common for patients with renal impairment. Overall, there was no significant difference in any-grade or Grade 3 or higher CRS and ICANS between the two groups. Those with renal impairment had a longer median hospital stay (13.5 vs. 9. *p* = 0.03). The renal impairment group had a higher percentage of cytopenias. Observations included more Grade 3 or higher anaemia and thrombocytopenia at Day 7, more Grade 3 or higher neutropenia, and thrombocytopenia at Day 30. Also, more Grade 3 or higher anaemia was observed by Day 60, but by Day 90 there were no significant difference in Grade 3 or higher cytopenias. Overall, there were no significant differences in mPFS and mOS between the RI and no RI groups. The mPFS in the RI vs. non-RI groups was 9 and 8 months, respectively. The median OS in the RI vs. non-RI groups was NR and 15.5 months [29].

This study highlighted the feasibility and safety of CAR-T therapy in RRMM and that efficacy was not impacted by renal impairment. However, it should be noted that there was a small number of patients in the RI group, particularly the very small number of patients with severe RI. Overall, such patients should not be excluded from being considered for CAR-T therapy.

### 7.2. Geriatric Population

Berdeja et al. [30] reported on subgroup analysis patients enrolled in the KarMMa trial [30]. The median PFS was 8.6 months (95% CI, 4.0–12.2) in patients aged 65 years older; and for those aged 70 years or more, the mPFS was 10.2 months (95% CI, 3.1–12.3). Overall survival data were not mature at the time of report and no new safety signals were observed. Overall, they reported deep and durable response with Ide-cel treatment together with a manageable safety profile in triple-class exposed patients with RRMM aged at least 65 years and 70 years [30].

A meta-analysis by Akhtar et al. [31] evaluated prospective clinical trials and observation studies of anti-BCMA CAR-T therapy in patients with MM. In total, 14 studies were included for data extraction, all of which were non-randomised trials, yielding a total of 5588 patients. A total of 26.2% (n = 146) were of older adults (at least 65 years of age). Overall, the response rates and rates of CRS in older adults are comparable to younger patients. However, there was an increased rate of ICANS (any grade) or neurotoxicity in older patients (15% in older adults compared with 6% in younger patients), though statistical significance could not be determined [31].

Reyes et al. [32] conducted a single-centre retrospective study evaluating the safety and efficacy of BCMA CAR-T cell therapy in those aged ≥70 years old at infusion versus those of the younger age group. In total, 83 participants had successfully received CAR-T therapy, either through standard of care or in a clinical trial. A total of 61 were aged <70 and 22 were aged ≥70. The older group were more likely to have a significantly lower creatinine clearance and more likely to have received reduced-dose lymphodepletion. There was also a slightly longer vein-to-vein interval in the older patients compared to younger patients at 45.5 days vs. 40 days, respectively (*p* = 0.04). The incidences of CRS and ICANS were comparable between the older and younger patients: 77% vs. 78% for CRS and 9% vs. 13% for ICANS. The median time to neutrophil count recovery after CAR-T infusion was also similar: 12.5 days vs. 13 days, respectively. Infection within 6 months was not significantly different between the two groups, though the numbers were small. Delirium or falls within 6 months were also not significantly different between the two groups, though these numbers were very small. The overall response rate was similar, with the older age group at 82% and 89% in the younger age group. Achievement of a CR between the older and younger group was 59% vs. 54%, respectively. MRD negativity was also not significantly different, with 50% in the older age group vs. 59% in the younger age group. Median PFS was 13.1 months in older age group vs. 12.5 months in the younger age group. This was not significantly different, with a *p*-value of 0.42. Overall survival was also not significantly different between the two groups after adjusting for high-risk cytogenetics or bone marrow plasma cell burden [32].

These findings support that CAR T for MM in the older population is feasible, and that chronological age alone should not be a reason to withhold BCMA CAR-T therapy.

### 7.3. Prior BCMA-Targeted Therapy

In Hansen et al.’s real-world report on Ide-cel, subgroup analysis showed that prior BCMA-targeted therapy was associated with an inferior PFS (median PFS of 9.0 vs. 3.2 months in those who did not receive vs. received treatment, respectively) [15].

Subsequently, Ferreri et al. [33] conducted a retrospective multicentre observational study of patients with RRMM whom received commercial Ide-cel at 1 of the 11 US medical centres and evaluated the outcomes of patients who had received prior BCMA-TT. Of the 203 patients who were infused with Ide-cel, 50 had prior BCMA-TT whilst 153 did not. Of the patients who received prior BCMA-TT, 76% received antibody–drug conjugate—primarily commercially available belantamab mafodotin. In total, 14% had prior bispecific antibody and 10% had prior CAR T, with all prior bispecific antibody and CAR T in clinical trials. The median PFS in those who received prior BCMA-TT was 3.2 vs. 9.0 without prior BCMA-TT. The 6-month median OS was 72% in prior BCMA-TT versus 89% in the group without prior BCMA-TT. However, it should be noted that the prior BCMA-TT group had a higher percentage of penta-refractory patients and more median prior lines of therapy [33].

Cohen et al. [34] reported on the outcomes of cohort C from the CARTITUDE-2 trial. This was a phase II multicohort, open-label study evaluating safety and efficacy in several patient populations, and in this report, they reported on enrolled quadruple-class exposed patients who had prior exposure to non-cellular BCMA-TT. Of the 24 patients enrolled and apheresed, 20 were treated with Cilta-cel. A total of 13 patients received the antibody–drug conjugate belantamab, whilst 7 received a bispecific antibody. At a median follow-up of 11.3 months, the ORR was 60% with 55% achieving at least a VGPR and 30% achieving at least a CR. The median PFS was 9.1 months and the median OS was not reached. In patients in the anti-BCMA ADC exposed group with a median follow-up of 11.8 months, the median PFS was 9.5 months. Of those who received the bispecific antibody, the mPFS was 5.3 months [34]. This suggests that sequencing Cilta-cel after prior BCMA-targeted therapy may still offer a meaningful clinical benefit, though the numbers are small in this cohort.

## 8. Challenges and Considerations for Canada

Although CAR T-cell therapy is likely to help improve the outcomes for RRMM patients in Canada, several challenges are anticipated when CAR T-cell therapy for MM becomes approved in Canada (see Table 3).

### 8.1. Patient Access—Institutional Factors

The delivery of CAR T-cell therapy is restricted to highly specialized centres. This is because administration and patient management require highly-specialized, trained staff and are resource-intensive. Patients require adequate monitoring and management of adverse events which may require input from other specialties including infectious diseases, neurology, or intensive care units [12].

Firstly, this will add to the growing demand and indications for CAR-T therapy and will likely put further constraints on the infrastructure of each CAR-T therapy centre. Depending on staffing levels and the availability of apheresis units and hospital beds, there will be a limited number of treatment slots per month at each centre [12,35]. Significant infrastructure planning and investment would be required to expand the capacity at each centre.

Secondly, there may be access disadvantages for patients living further away from these centres, particularly if they live in remote areas, or from different cities or other provinces. Even in the setting of an allogeneic stem cell transplant (alloSCT) where similar barriers to treatment exist, Morakinyo et al. have reported significant differences in transplant rates by provincial residence in Canada [36]. As mentioned before, for at least the first 30 days, there is a need to reside within a specified travel distance to the treatment centre to ensure safety and timely access to specialized healthcare post CAR T-cell infusion. The cumulative costs of travel and temporary accommodation particularly for those who live further away may be a significant barrier [12,37]. Fortunately, CAR-T companies often have a patient assistance program that may help to alleviate these costs, though the inclusion criteria for such programs vary from one company to another.

Adding to this barrier, there is a requirement to have a dedicated caregiver for at least the first 30 days post-infusion. Paid caregiving is economically inaccessible to many; hence, patients often need to rely on family or friends who would be uncompensated financially. Those who live further away or are socially isolated may have difficulty finding a suitable caregiver. Transitional care facilities would be an option for these patients.

There are also concerns that ethnic minorities may be disadvantaged in access to CAR T-cell therapy. In the US, Ahmed et al. reported that African-Americans were less likely than any other racial/ethnic groups to receive CAR T-cell therapy. Furthermore, African-Americans and Hispanics were underrepresented in clinical trials for which the reasons are multifactorial [37]. However, it has been postulated that Canada’s universal health care insurance program may mitigate against the socioeconomic barriers that impact healthcare access. Encouragingly, Morankinyo et al. reported that indigenous patients, ethnic minorities, and those with a low income status were not associated with lower alloSCT rates in Canada [36]. However, active surveillance of CAR-T therapy rates in Canada will be required to determine if disparities exist.

It would be important for the treating CAR-T facilities to help patients and their caregivers navigate these complexities. Early referral to a social worker should also be considered. Provincial cancer agencies must work with regional and local sites to ensure adequate supports are in place for equitable access to CAR-T therapy for myeloma patients in Canada.

### 8.2. Patient Access—Manufacturing Factors

CAR T-cell manufacturing is a complex process and thus, the current industry model is of highly centralised CAR T-cell manufacturing sites. Therefore, the patient’s apheresed T cells must be transported, often internationally to the manufacturing facility, to be genetically engineered to express the chimeric antigen receptor, then finally shipped back to the administrating facility to be infused to the patient. This entire process is logistically complex, costly, and more importantly adds to the vein-to-vein time which can be up to six weeks [12,38]. Patients often have progressive disease during this period and require bridging therapy which may lead to increased toxicity [12]. Not all patients who have been leukapheresed would then receive their CAR T-cell therapy. There can be manufacturing failures or the patient may have rapidly progressive disease or disease-related mortality. In a real-world report of Cilta-cel, 38 (21%) out of 177 leukapheresed patients did not receive Cilta-cel [20].

The technical challenges in CAR T-cell manufacturing including reagent supply (such as viral vector shortages) and stringent requirements for “in-specification” products also limit the timely delivery of products. As a result, there are a limited number of CAR-T slots that can be allocated to each treatment centre. Kourelis et al. conducted a survey on 20 CAR-T centres across the US. Of the 17 who responded, a median of 1 Ide-cel slot was allocated per month per centre and 15 centres were allocated ≤2 slots per month (range 0–4). The median number of patients per centre on the waitlist since Ide-cel approval was 20 per month. As a result, patients remained on the waitlist for a median of 6 months prior to leukapheresis. The responders estimated 25% of patients on this waitlist had died or enrolled in hospice [39]. Thus far, this has not been the experience in Canada. However, as the immune effector cell programs expand in each province, there will be a need to increase manufacturing capacity. As such, foreplanning and a constant dialogue between the CAR T-cell centres and the manufacturing providers will be required to ensure the manufacturing bottlenecks described in the US are not repeated here.

### 8.3. Cost to the Public Sector

Anti-BCMA CAR T-cell therapy remains the most expensive myeloma treatment to date [40]. The cost for one infusion ranges from USD 419,500 (Ide-cel) to USD 465,000 (Cilta-cel). This does not yet factor in other costs including hospital stay and infection prophylaxis (particularly with intravenous immunoglobulins) [12,38]. The public health care system must be able to cover all of these costs, particularly with the expanded indication for CAR T-cell therapy, otherwise hospitals administering CAR T-cell therapy may then limit their immune effector cell therapy programs if they are unable to be fully reimbursed [12].

Furthermore, the high cost to the public sector must be matched by value for money [41]. Patients who have achieved a durable response from CAR-T therapy are off other myeloma therapy or maintenance therapy which are typically given continuously until significant toxicity or relapse [40]. In these patients, savings may be found with the one-off CAR T-cell therapy against the cumulative cost of other myeloma therapy. However, in both Cilta-cel and Ide-cel, a significant proportion of patients do not derive a long-term benefit [15,18]. Cost-effectiveness would be dependent on the mPFS, and longer-term outcomes of anti-BCMA CAR-T trials would be required to fully determine the cost-efficacy in a publicly funded healthcare setting [40].

Treatment cost-effectiveness may also depend on the physician’s ability to better prospectively distinguish patients who are likely to achieve a durable response versus those who are not, particularly if there were modifiable factors prior to leukapheresis and CAR T-cell manufacturing [41]. This is certainly an area of active research [42]. Gagelman et al. [43] recently proposed the MyCARe model, a simple scoring system incorporating disease, treatment, and inflammation-related variables to stratify patients into those at a low, intermediate, and high risk of early treatment failure (defined as relapse or progression within 5 months from infusion) [43]. This prognostic model has been externally validated and maintains prognostic utility across different CAR-T products, treatment regions (Europe and the US), and penta-refractory status [43]. The MyCARe model may assist with patient selection and the optimal timing of CAR T-cell therapy in patient-specific subgroups, [43]. However, it should not be used to exclude patients from such therapy.

There is interest among the academic centres to develop the in-house manufacturing of CAR-T products through closed benchtop systems. A similar model has been employed successfully in Spain [12,41]. Switzerland has already started decentralised manufacturing and is able to provide CAR T-cell therapy at a significantly reduced cost and time to CAR T-cell infusion [44].

### 8.4. Impact on Community Hospitals and Acute Care Services

Given the logistical complexities of CAR T-cell therapy and likely limited treatment slots per month at each CAR-T centre, early inquiries or referrals from community providers should be encouraged. Naturally, the process from referral to CAR T-cell infusion is likely to take more than 6 weeks; thus, close communication between the referring centre and CAR-T centre should be maintained throughout. After baseline evaluation, there is a washout period for anti-myeloma therapies to ensure T-cell fitness prior to leukapheresis. During the 4–6-week manufacturing period, the most appropriate bridging therapy should be made on a case-by-case basis between the referring physician and the CAR-T provider. The bridging therapy may be provided by the referring centre. Once the CAR-T product is ready, close coordination is required to ensure adequate washout, availability of a hospital bed at CAR-T centre, and timely release of the product for infusion [45].

As mentioned above, patients who have received CAR T-cell therapy are prone to opportunistic infections, have prolonged cytopenias, or develop late neurotoxicity. These patients will eventually return to their local hospital for long-term care with some oversight from the CAR T-cell therapy centre. Local providers and acute care services will also need education and training on the recognition and management of prolonged or late adverse events associated with CAR T-cell recipients. Transfusion support and immunoglobulins may be required. Lastly, the long-term monitoring of the patient’s myeloma will still fall upon the patient’s primary physician at their local hospital, as CAR T-cell therapy is not curative and relapses may still occur. The sequencing of therapies before and after CAR T-cell therapy is still under investigation. BiTE and other novel emerging agents may be considered for relapse(s) after CAR T-cell therapy.

## 9. Future Directions

Multiple trials are underway examining the use of Ide-cel and Cilta-cel earlier in the disease course. Two published landmark trials should be mentioned which indicate the earlier use of CAR T-cell therapy.

CARTITUDE-4 is a phase III, randomized, open-label trial comparing patients with lenalidomide-refractory MM to receive Cilta-cel or the physician’s choice of effective standard of care therapy (pomalidomide, bortezomib, and dexamethasone; or daratumumab, pomalidomide, and dexamethasone). All patients had received one to three prior lines of therapy. A total of 419 patients underwent randomization (208 received Cilta-cel and 211 received the standard of care). At a median follow-up of 15.9 months, the median PFS was not reached in the Cilta-cel group but was 11.8 months in the standard of care group. The PFS at 12 months was 75.9% in the Cilta-cel group and 48.6% in the standard of care group. Death from any cause was reported in 39 patients in the Cilta-cel group and 46 in the standard of care group (HR, 0.78; 95% CI, 0.5–1.2) [46]. Based on this study, on 5 April 2024, the FDA approved Cilta-cel for RRMM in patients who have received at least one prior line of therapy, including a PI and an IMiD and who are refractory to lenalidomide [47].

KarMMA-3 is also an international, phase III, open-label trial comparing adults with RRMM who have received 2–4 regimens previously (including IMiD, PI, and daratumumab), disease refractory to last regimen, and randomized to either Ide-cel or one of five standard regimens. At a median follow-up of 18.6 months, the median PFS was 13.3 months in the Ide-cel group as compared with 4.4 months in the standard regimen group. Data on overall survival were immature [48]. As of the 4 April 2024, the FDA has approved Ide-cel for patients with RRMM after two or more prior lines of therapy including an IMiD, PI, and anti-CD38 monoclonal antibody [49].

Trials are also underway investigating the use of Ide-cel and Cilta-cel for frontline therapy in those with newly diagnosed multiple myeloma (NDMM). The KarMMA-4 trial is a phase I study investigating Ide-cel in patients with high-risk NDMM. The CARTITUDE-5 trial is a phase III randomised clinical trial studying the efficacy of Cilta-cel in patients with transplant-ineligible NDMM. CARTITUDE-6 is the first randomised clinical trial comparing CAR T-cell therapy against an autologous stem cell transplant (ASCT) in NDMM. Long-term follow-up and cost-efficacy studies would be required to determine whether ASCT would be fully replaced by CAR T-cell therapy [50].

Other CAR T-cell therapy products for RRMM are under development. The UNIVERSAL trial is a phase I study investigating the feasibility of ALLO-715, a first-in-class, allogeneic, anti-BCMA CAR T-cell therapy in patients with RRMM and who have had at least three prior lines of therapy (including a PI, IMiD, or anti-CD38 monoclonal antibody). The advantage of allogeneic CAR T-cell therapy is that it is an off-the-shelf product and can overcome the manufacturing wait time in autologous CAR T-cell therapy. Certainly, in this study, the median time from enrolment to start of lymphodepletion was 5 days [51]. Of the 43 patients, CRS was observed in 56% and neurotoxicity in 14%, with only one case of Grade 3 CRS and no Grade ≥ 3 neurotoxicity. No GVHD was reported. Therefore, this initial report supports the safety and feasibility of allogeneic CAR T-cell therapy in multiple myeloma.

There are also non-BCMA-targeting CAR T-cell therapies for MM in development. MCARH109 is a GPRC5D-targeting CAR T-cell therapy. In the phase I dose escalation trial, of the 17 patients, a response was reported in 71%. Of those who had received previous BCMA therapies, responses were observed in 7 out of 10 such patients.

Finally, newer generations of CAR constructs are under investigation to improve the immunogenicity, efficacy, and persistence of CAR T cells, as well as reducing unwanted side effects [52].

## 10. Conclusions

CAR T-cell therapy will soon be available as the standard of care for Canadians with RRMM. This addition to the current standard of care therapies in Canada is likely to significantly improve survival in patients with multiple myeloma. The optimal sequence of CAR T-cell therapy amongst other available therapies is likely to be important. Strategies must be in place to ensure equitable access to immune effector cell therapy for Canadians with multiple myeloma. Although CAR T-cell therapy for multiple myeloma would be delivered in a limited number of highly specialized centres, haematologists from referring centres will still play an important role in the care of patients with RRMM. Early referral to a CAR T-cell therapy centre is essential in order to expedite the time to treatment. Patients’ haematologists from their local hospital will still play an important role in the ongoing monitoring and management of opportunistic infections, prolonged cytopenias, and late neurotoxicity. Many challenges remain in CAR T-cell therapy for multiple myeloma; thus, ongoing research and development is required to overcome these hurdles.

## Figures and Tables

**Table 1:** Major studies on outcomes of patients with RRMM without CAR T-cell therapy or a bispecific antibody.

	MaMMOTH [6]	LocoMMotion [4]	CMRG [5]
Study design	Retrospective cohort	Prospective observational	Retrospective cohort
Institutions	14 academic centres in the US	76 sites, Europe and US	17 academic centres, Canada
Study period	January 2017–June 2018	August 2019–October 2020. Data cutoff: May 2021.	2007–June 2022.
Patient numbers	*N* = 275	*N* = 248	*N* = 346
Primary population	Refractory to anti-CD38 monoclonal antibody	Received at least 3 prior lines of therapy, or were double-refractory to a PI and an IMiD, or triple-refractory to PI, IMiD, and anti-CD38 monoclonal antibody, and have documented progression during or after their last line of therapy.	Refractory to anti-CD38 monoclonal antibody based regimen and subsequently treated with standard of care regimen.
Median prior lines of therapy	4 (range 1–16)	4 (range 2–13)	3 (range 1–9)
Triple-class and penta-refractory	Triple-class: 54% ** Penta-refractory: 25%	Triple-class: 73.8% Penta-refractory: 17.7%	Triple-class: 58%
Median follow-up	10.6 months (range 1.0–42.3 months	11.01 months (range 0.1–19.2)	8 months
**Outcomes**			
Overall ORR *	47%	29.8% (95% CI: 24.2–36.0)	48%
Overall mPFS	3.4 months (95% CI 2.8–4.0)	4.6 months (95% CI 3.9–5.6)	4.6 months (95% CI 4.1–5.6)
Overall mOS	8.6 months (95% CI 7.2–9.9)	12.4 months (95% CI: 10.28–NE)	13.3 months (95% CI: 10.6–16.6)
Triple-refractory * ORR	29% **	25.1% (95%CI: 19.0–32.1)	40%
Triple-refractory mPFS	NR	3.9 months (95% CI 3.4–4.6)	4.4 months (95% CI 3.6–5.3)
Triple-refractory mOS	9.2 months (95% CI 7.1–11.2)	11.1 months (95% CI 88–14.2)	10.5 months (95% CI 8.5–13.8)

ORR: overall response rate. mPFS: median progression free survival. mOS: median overall survival. NR: not reported. * Response to subsequent line of therapy. ** Triple- or quad-refractory.

**Table 2:** Summary of Ide-cel and Cilta-cel pivotal trials and real-world studies.

	KarMMA [14]	Myeloma CAR-T Consortium [15]	CIBMTR Registry [16]	CARTITUDE-1 [17,18,19]	Hansen et al. [20]
CAR T-cell therapy product	Ide-cel	Ide-cel	Ide-cel	Cilta-cel	Cilta-cel
Study design	Phase II (pivotal)	Retrospective cohort (Real world)	Observational (Real world)	Phase 1b/II (pivotal)	Retrospective cohort (Real world)
Institutions	20 institutions 7 countries (US, Canada, Europe)	11 US institutions	US institutions	16 US institutions	12 US institutions
Participant primary characteristics	Received at least 3 prior regimens including IMiD, PI and anti-CD38 antibody ECOG 0–1 Measurable disease	RRMM who had received at least 4 prior lines of therapy and underwent leukapheresis from 1 April 2021–28 February 2022.	First 603 adult patients received commercial Ide-cel and reported to CIBMTR registry	Received 3 or more prior lines of therapy or become double-refractory to PI and IMiD, and have received PI, IMiD, and anti-CD38 antibody	Adults treated with intended standard of care Cilta-cel up to the data cutoff 31 December 2022
Dose of CAR+ T cells	150 × 10^6^ in 3% 300 × 10^6^ in 55% 450 × 10^6^ in 42%	<400 × 10^6^ in 41% >400 × 10^6^ in 59%	<400 × 10^6^ in 44% >400 × 10^6^ in 56%	0.75 × 10^6^/Kg	0.6 × 10^6^/Kg (range: 0.1–0.9)
Patients leukapheresed	140	196	NR	113	177
Patients infused *	128	159	603	97	139
Median age (range)	61 (33–78)	64 (36–83)	65	61 (43–78)	64
Median prior lines	6 (3–16)	4	7 (4–21)	5	6 (2–18)
HR Cytogenetics %	35	38	23	27	41
Extramedullary disease %	39	47	17	10	35
Triple-refractory %	84	84	NR	86	NR
Penta refractory %	26	44	36	42	36
Median follow-up	13.3 months	6.1 months	6.6 months	33.4 months	2.3 months (0–8)
**Outcomes**					
Overall response rates (ORR) %	73	84	71	97	80
Complete response rates (CR) %	33	42	27	67 (stringent CR)	40
Median PFS (months)	8.8 (95% CI 5.6–11.6)	8.5 (95% CI 6.5 to NE)	Short follow-up PFS at 6 months: 62%	34.9 (95% CI 25.2–NE)	Short follow-up NR
Median OS (months)	19.4 ** (95% CI 18.2–NE)	12.5 (95% CI 11.3 to NE)	Short follow-up OS at 6 months: 82%	Not reached At 36 months: 62.9% **	Short follow-up NR
**Adverse events**					
Any grade CRS %	84	82	81	95	81
Grade 3+ CRS %	5	3	3	4	7
Median time to CRS (days)	1	NR	2	7	NR
Any grade neurotoxicity %	18	18	27	21	22
Grade 3+ Neurotoxicity %	3	6	4	9	8
Median time to neurotoxicity (days)	2	NR	2	8	NR

HR: high risk. PFS: progression free survival. OS: overall survival. CRS: cytokine release syndrome. NR: not reported. * denominator for outcome analysis. ** estimated.

**Table 3:** Summary of challenges and potential solutions for myeloma CAR T-cell therapy in Canada.

Challenges	Potential Solutions
Limited treatment slots at the CAR-T centre	Foreplanning and infrastructure investment to expand CAR-T programs; Includes increasing specialised staff, apheresis units, and hospital beds
Cost of travel and temporary accommodation	CAR-T company’s patient assistance programs; Early referral to social worker
Unable to find suitable caregiver	Transitional care facilities
Inequitable access for certain groups	Canada’s universal health care insurance program; Active surveillance by provincial cancer agencies
Long vein to vein time	Patient selection; Bridging therapy; Investment for CAR-T manufacturing in Canada/Decentralised manufacturing
Manufacturing bottleneck	Increase manufacturing capacity—requires foreplanning and close dialogue between CAR T cell centres and manufacturing providers
High cost to public sector	In-house manufacturing at academic centres or decentralised manufacturing; Patient selection
Complex ongoing care post CAR-T therapy	Education and training on recognition and management of prolonged or late adverse events; Ongoing research
